# Influence of temperature on the δ^13^C values and distribution of methanotroph‐related hopanoids in *Sphagnum*‐dominated peat bogs

**DOI:** 10.1111/gbi.12389

**Published:** 2020-03-16

**Authors:** Julia F. van Winden, Helen M. Talbot, Gert‐Jan Reichart, Niall P. McNamara, Albert Benthien, Jaap S. Sinninghe Damsté

**Affiliations:** ^1^ Department of Earth Sciences Faculty of Geosciences Utrecht University Utrecht The Netherlands; ^2^ School of Civil Engineering and Geoscience Newcastle University Newcastle upon Tyne UK; ^3^ Departments of Marine Microbiology and Biogeochemistry and Ocean Systems NIOZ Royal Netherlands Institute for Sea Research, and Utrecht University Den Burg The Netherlands; ^4^ UK Centre for Ecology & Hydrology Lancaster Environment Centre Lancaster UK; ^5^ Alfred Wegener Institute, Helmholtz Centre for Polar and Marine Research Bremerhaven Germany; ^6^ Present address: Department of Archaeology (BioArCh) University of York York UK

**Keywords:** bacteriohopanepolyol, carbon isotopes, methane, methanotroph, *Sphagnum*, temperature

## Abstract

Methane emissions from peat bogs are mitigated by methanotrophs, which live in symbiosis with peat moss (e.g. *Sphagnum*). Here, we investigate the influence of temperature and resultant changes in methane fluxes on *Sphagnum* and methanotroph‐related biomarkers, evaluating their potential as proxies in ancient bogs. A pulse‐chase experiment using ^13^C‐labelled methane in the field clearly showed label uptake in diploptene, a biomarker for methanotrophs, demonstrating in situ methanotrophic activity in *Sphagnum* under natural conditions. Peat cores containing live *Sphagnum* were incubated at 5, 10, 15, 20 and 25°C for two months, causing differences in net methane fluxes. The natural δ^13^C values of diploptene extracted from *Sphagnum* showed a strong correlation with temperature and methane production. The δ^13^C values ranged from −34‰ at 5°C to −41‰ at 25°C*.* These results are best explained by enhanced expression of the methanotrophic enzymatic isotope effect at higher methane concentrations. Hence, δ^13^C values of diploptene, or its diagenetic products, potentially provide a useful tool to assess methanotrophic activity in past environments. Increased methane fluxes towards *Sphagnum* did not affect δ^13^C values of bulk *Sphagnum* and its specific marker, the C_23_ *n*‐alkane. The concentration of methanotroph‐specific bacteriohopanepolyols (BHPs), aminobacteriohopanetetrol (aminotetrol, characteristic for type II and to a lesser extent type I methanotrophs) and aminobacteriohopanepentol (aminopentol, a marker for type I methanotrophs) showed a non‐linear response to increased methane fluxes, with relatively high abundances at 25°C compared to those at 20°C or below. Aminotetrol was more abundant than aminopentol, in contrast to similar abundances of aminotetrol and aminopentol in fresh *Sphagnum*. This probably indicates that type II methanotrophs became prevalent under the experimental conditions relative to type I methanotrophs. Even though BHP concentrations may not directly reflect bacterial activity, they may provide insight into the presence of different types of methanotrophs.

## 1 INTRODUCTION

Peat bogs play an important role in the global carbon cycle since they sequester one‐third of the Earth's terrestrial carbon (Smith et al., 2004). Peat bogs are responsible for approximately 10% of the total methane flux to the atmosphere, having a major impact on atmospheric methane concentrations (Gorham, 1991). High latitudes are especially vulnerable to climate change, and it is, therefore, important to understand the effect of changing environmental conditions on carbon and net methane flux rates in peat bogs.

Aerobic methane oxidation by bacteria (methanotrophs) is a significant terrestrial methane sink and hence plays an important role in the global methane cycle (Dean et al., 2018; Hanson & Hanson, 1996). In peat bogs, methanotrophs live in symbiosis with peat moss (*Sphagnum*)*,* living inside the hyaline cells of *Sphagnum* and on the stems. There they reduce methane emissions and provide CO_2_ and fixed nitrogen to *Sphagnum* when growing under submerged conditions (Kip et al., 2010; Larmola et al., 2010, 2014; Raghoebarsing et al., 2005). In order to predict the consequences of perturbations in the global carbon cycle, a better understanding of the influence of environmental factors on methanotrophs and the methane cycle in peat bogs is essential. Mesocosm experiments have revealed that both methane production and oxidation increase with increasing temperature, albeit that the extent of methane retention by the *Sphagnum*‐methanotroph consortium strongly decreases with increasing temperature (van Winden, Reichart, McNamara, Benthien, & Sinninghe Damsté, 2012). This confirmed the hypothesis that peat bogs may act as a positive feedback to global warming. Assessing the long‐term impact of changing environmental conditions on methanotrophs and the methane cycle in peat bogs, however, also relies on reconstructions of methane fluxes in ancient peats.

Ombrotrophic peat bogs receive their nutrients solely via precipitation, which makes them relatively nutrient poor. The low pH buffering capacity, cation exchange and humic acid release by *Sphagnum* mosses result in a strongly acidic environment (Soudzilovskaia et al., 2010, and references therein). Due to the low pH of the bog water, the reservoir of exchangeable inorganic carbon is low and the level of diffusion of atmospheric CO_2_ into the bog water is also little, which makes submerged‐growing *Sphagnum* mosses CO_2_‐limited and to a large extent dependent on CO_2_ derived from organic matter degradation (Smolders, Tomassen, Pijnappel, Lamers, & Roelofs, 2001). Methanotrophs supply methane‐derived CO_2_, which may account up to 35% of the carbon uptake by *Sphagnum* (Kip et al., 2010; Larmola et al., 2010).

The knowledge on environmental controls, including temperature, on methanotrophy is still limited. If palaeoclimate archives could be used to study the controls on methane fluxes in peat bogs, including temperature and humidity, this would open up additional sources of information. While advanced microbial techniques exist to study current microbial community structures (see Yates, Nakatsu, Miller, & Pillai, 2016), the limited longevity of genetic material, however, limits the use of advanced microbial tools to reconstruct changes through time. Changes in past methanotrophic activity in peat bogs can potentially be studied using hopanoids. These bacterial markers are common lipids of methanotroph, also those occurring in *Sphagnum* peats (van Winden, Talbot, Kip, et al., 2012). However, they are not exclusive to methanotrophs since many other bacteria produce them as well (Belin et al., 2018; Farrimond et al., 1998; Rohmer, Bouvier‐Nave, & Ourisson, 1984; Sinninghe Damsté, Rijpstra, Dedysh, Foesel, & Villanueva, 2017; Sinninghe Damsté et al., 2004). Bacteriohopanepolyols (BHPs) have a polyfunctionalized side chain attached to the pentacyclic carbon skeleton, to which a variety of polar groups can be attached (Talbot & Farrimond, 2007). Differences in these polar groups hold species‐specific information (Ourisson, Rohmer, & Poralla, 1987; Rohmer et al., 1984; Rush et al., 2016), and therefore, BHPs may provide insight into methanotroph community structures (Coolen et al., 2008; van Winden, Talbot, Kip, et al., 2012). Methanotrophs occurring in peat bogs can be generally divided into two distinct phylogenetic groups: γ‐proteobacteria (type I) and α‐proteobacteria (type II; Esson et al., 2016; Hanson & Hanson, 1996). Characteristic biomarkers for type I methanotrophs are aminobacteriohopanepentols (aminopentol), while aminobacteriohopanetetrol (aminotetrol) is primarily produced by type II methanotrophs, but also to a minor extent by type I methanotrophs and some sulphate‐reducing bacteria (Blumenberg et al., 2006; Coolen et al., 2008; Cvejic, Bodrossy, Kovács, & Rohmer, 2000; Neunlist & Rohmer, 1985a, 1985b; Osborne et al., 2017; Rohmer et al., 1984; Rush et al., 2016; Talbot, McClymont, Inglis, Evershed, & Pancost, 2016; Talbot, Watson, Murell, Carter, & Farrimond, 2001; van Winden, Talbot, Kip, et al., 2012).

Methanotroph lipids are typically severely depleted in ^13^C since biogenic methane has relatively depleted δ^13^C values, ranging from approximately −45‰ to −65‰ (Hornibrook, Longstafe, & Fyfe, 2000), and this methane acts as a carbon source. Therefore, these lipids serve as an indicator for methanotrophy as shown for lakes and marine settings (Collister, Summons, Lichtfouse, & Hayes, 1992; Coolen et al., 2008; Elvert, Whiticar, & Suess, 2001; Freeman, Hayes, Trendel, & Albrecht, 1990; Pancost & Sinninghe Damsté, 2003; Spooner et al., 1994). However, hopanoids in *Sphagnum* and other peats often show only a limited depletion in ^13^C (see Inglis, Naafs, Zheng, Schellekens, and Pancost (2019) for a comprehensive overview). For example, van Winden et al. (2010) reported δ^13^C values ranging from −31‰ to −38‰ for hopenes (hop‐17(21)‐ene + 2‐methylhop‐17(21)‐ene) and −34‰ to −37‰ for 17β,21β(H)‐bishomohopanol (derived from tetrafunctionalized hopanoids) in *Sphagnum* moss species from contemporary peatlands. Pancost, van Geel, Baas, and Sinninghe Damsté (2000) also reported only non‐depleted δ^13^C values for hopanoids in contemporary peatlands, ranging from −27‰ to −30‰ for 17β,21β(H)‐bisohomohopanol and from −22‰ and −27‰ for 17α,21β(H)‐homohopane. Huang et al. (2018) observed δ^13^C values between −28‰ and −40‰ found for C_29_ 17β,21β(H)‐norhopane in a peatland in Central China. Zheng et al. (2014) reported δ^13^C values as low as to −50‰ for diploptene in a *Carex*‐dominated Holocene peat deposit from the Tibetan Plateau. In addition, in the Cobham lignite, a Paleogene wetland deposit, δ^13^C values down to −42‰ and −76‰ were observed for C_31_ and C_29_ 17β,21β(H)‐hopanes, respectively (Pancost et al., 2007). These depleted isotope signatures were interpreted to be indicative of increased methanotrophic activity. In our previous study, *Sphagnum* from settings with highest methane oxidation rates exhibited the most depleted hopene δ^13^C values, in contrast to δ^13^C values of the derived 17β,21β(H)‐bisohomohopanol, and therefore, δ^13^C values of hopenes probably reflect methanotrophic activity (van Winden et al., 2010).

In this study, we investigate the influence of temperature and the resultant changes in methane flux rates on *Sphagnum* and methanotroph‐related hopanoids, evaluating their potential as proxies for methanotrophs and methane fluxes in ancient bogs. To this end, *Sphagnum* mosses, incubated at different temperatures in a previously described mesocosm experiment (van Winden, Reichart, et al., 2012), were analysed for their compound‐specific carbon isotopic composition and BHP composition. These parameters were subsequently compared to methane flux rates, to evaluate their potential as proxies for methane fluxes.

## 2 MATERIALS AND METHODS

### 2.1 Site description

The field site used in this study was in Moorhouse Nature Reserve in the North Pennines, UK, and the site has been described in detail elsewhere (McNamara et al., 2008 and references therein). Briefly, Moorhouse is an acidic (pH 3.0–4.2), ombrotrophic blanket bog, with numerous gullies cutting through the blanket. The vegetation on the blanket is typical for hummock peat, containing *Calluna vulgaris, Eriophorum vaginatum, Eriophorum angustifolium, Pleurozium schreberi* and *Sphagnum capillifolium*. In waterlogged areas, pools or gullies, vegetation was dominated by *Sphagnum cuspidatum*, *E. angustifolium* and *E. vaginatum*. Average rainfall is high at 1,900 mm per year. Our research was performed in a wide gully with limited lateral flow, dominated by *S. cuspidatum,* with the top of the decomposed peat approximately 15 cm below the water table.

### 2.2 Field pulse‐chase experiment

Large PVC tubes (height 1 m; width: 23.5 cm) were inserted into the peat, to prevent lateral migration of labelled methane to be injected. This was done six weeks prior to the in situ experiments to allow the environment to recover. *Eriophorum* was removed from the study site, to examine the *Sphagnum*‐associated methanotrophs exclusively. We injected 100 ml demineralized water, saturated with 99 atom% ^13^C‐labelled methane, into the peat within the PVC tubes at 40 cm depth, roughly corresponding to a depth where methane is typically produced (Hornibrook, Bowes, Culbert, & Gallego‐Sala, 2009). Injection was performed using a 100‐ml syringe attached to a thin metal rod (1 mm inner diameter) via six 1 mm openings on the sides at the base of the rod. *Sphagnum* was harvested from parallel tubes at days 2, 6, 11 and 14 consecutively, and sectioned into top, middle and lower parts. Lower parts were analysed for the ^13^C values of the diploptene.

### 2.3 Temperature experiments

Transparent cores (height 50 cm, diameter 7 cm), containing approximately 30 cm of peat with on top living *S. cuspidatum,* were taken from the field site in July 2009, when temperatures ranged between 10 and 20°C. These peat cores were incubated at 5, 10, 15, 20 and 25°C in triplicate at the Alfred Wegener Institute (AWI) in Bremerhaven for two months. The lower 30 cm of the cores, consisting of decomposed peat below the living *Sphagnum*, were covered with aluminium foil and sealed with rubber stoppers. Since peat has a very large redox buffering capacity, no amendments were done to make the water anaerobic or acidic. *Eriophorum* plants were not included in the incubation. Water levels were kept at the level of the top of the *Sphagnum* capitula, which increased in height with growth. Water level was kept constantly high by addition of bog water originating from the field site on a regular basis (several times per week) to ensure the *Sphagnum* remained fully submerged.

*Sphagnum* is a very basic plant, growing from the top and decaying at the bottom. After two months, when the photosynthetic capitulum was newly grown, the whole *Sphagnum* plant was harvested and sectioned into top parts (capitulum) and lower parts. Top parts of *Sphagnum* mosses were analysed for the δ^13^C of *Sphagnum‐*derived C_23_ *n*‐alkane to capture only lipids grown under the experimental conditions. The lower parts, where methanotrophs reside (Raghoebarsing et al., 2005; van Winden, Reichart, et al., 2012; van Winden, Talbot, Kip, et al., 2012), were analysed for BHPs and δ^13^C of a bacterial lipid (diploptene). The bulk carbon isotopic composition was measured of both the top and lower parts of the *Sphagnum* mosses.

### 2.4 Extraction and derivatization of lipids

Total extracts of freeze‐dried *Sphagnum* mosses (whole plant) derived from the field experiment were obtained with an Accelerated Solvent Extractor (Dionex), using a mixture of DCM:MeOH (9:1, v/v). Top parts and lower parts of freeze‐dried *Sphagnum* mosses obtained from the temperature experiments were extracted using a modified Bligh and Dyer extraction procedure, to enable BHP analyses (Talbot, Rohmer, & Farrimond, 2007). An aliquot of the extract was separated into an apolar fraction and a polar fraction over an activated Al_2_O_3_ column using hexane:DCM (9:1 v/v) and DCM:MeOH (1:1 v/v). To purify samples for hopene analyses (lower *Sphagnum* parts), apolar fractions were treated by urea adduction. Apolar fractions were dissolved in 200 μl urea‐saturated methanol, 200 μl acetone and 200 μl hexane and shaken. After 30 min at −20°C, solvents were evaporated under a stream of nitrogen. The urea crystals, containing the adductable normal and *iso* alkanes, were washed with hexane three times. To obtain the non‐adductable branched and cyclic hydrocarbons (including the hopenes), the urea crystals were washed with hexane three times. The wash solvents were collected, dried under nitrogen and dissolved in hexane prior to GC analyses.

For BHP analyses, another aliquot of the total extract of the lower *Sphagnum* parts was acetylated using acetic anhydride and pyridine (1:1) at 50°C for 1 hr and left at room temperature overnight, after addition of the internal standard pregnanediol. The acetylated extract was dried at 50°C under a continuous nitrogen flow and dissolved in MeOH/propane‐2‐ol (60:40 v/v), prior to LC/MS analyses.

### 2.5 Gas chromatography (GC), GC/mass spectrometry (GC/MS) and GC‐isotope ratio monitoring mass spectrometry (IRMS), Elemental Analyzer (EA)‐IRMS

Hopene‐containing fractions were analysed on a gas chromatograph (HP 6890) equipped with a flame ionization detector (FID) set at constant pressure (100 kPa). A fused silica column (30 m × 0.32 mm i.d., film thickness 0.1 μm) coated with CP Sil‐5CB was used with helium as a carrier gas. Extracts were injected on‐column at 70°C. The temperature increased with 20°C/min to 130°C and 4°C/min to 320°C, followed by an isothermal hold for 20 min. Components were identified using gas chromatography–mass spectrometry (Thermo Trace GC Ultra), using the same column and temperature programme as for GC analyses.

Compound‐specific δ^13^C values were determined by isotope ratio monitoring–gas chromatography mass spectrometry (GC‐IRMS, Thermo Fisher Delta V), using the same column and temperature programme as for GC analyses. Carbon isotopic values are reported in the delta notation relative to the VPDB standard.

Bulk carbon isotopes were analysed on homogenized subsamples from the lower and upper parts of the *Sphagna*. To minimize the impact of isotopic differences between different plant parts about ten times, the required amount of carbon was weighed in tin sample cups. The CO_2_ produced was subsequently diluted with He before being introduced online into the mass spectrometer (Finnigan Delta plus). Accuracy and precision based on replicate analyses of samples and an in‐house standard (calibrated to VPDB using international standards) were <0.1‰.

### 2.6 High‐performance liquid chromatography–atmospheric pressure chemical ionization mass spectrometry (HPLC/APCI‐MS)

Acetylated total extracts were analysed for BHPs by reversed‐phase high‐performance liquid chromatography (HPLC). The derivatization and analytical procedure have been described previously (Cooke, Talbot, & Farrimond, 2008). In short, reversed‐phase HPLC analysis was carried out using a Surveyor HPLC system (ThermoFinnigan) fitted with a Gemini (Phenomenex) C_18_ 5 μm HPLC column (150 mm, 3.0 mm i.d.) and a security guard column of the same material. Separation was achieved at a flow rate of 0.5 ml/min at 30°C with the following gradient profile: 90% A and 10% B (0 min); 59% A, 1% B and 40% C (at 25 min), then isocratic (to 40 min) and returning to the starting conditions over 5 min and finally stabilizing for 15 min before the next injection, where A = MeOH, B = water and C = propan‐2‐ol (all Fisher Scientific HPLC grade). LC/MS was performed using a ThermoFinnigan LCQ ion trap mass spectrometer equipped with an APCI source operated in positive ion mode. LC/MS analysis was carried out in data‐dependent mode with three scan events: SCAN 1—full mass spectrum, range *m/z* 300–1,300; SCAN 2—data‐dependent MS^2^ spectrum of most abundant ion from SCAN 1; and SCAN 3—data‐dependent MS^3^ spectrum of most abundant ion from SCAN 2. Quantification was performed using *m/z* traces targeting the characteristic base peak ions and calculated using averaged response factors for a limited suite of nitrogen and non‐nitrogen containing BHP standards where N‐containing BHPs give an averaged response 12 times that of the internal standard and those with no nitrogen 8 times that of the standard (Cooke et al., 2008). Accuracy of the quantification was ±20%.

## 3 RESULTS

**Figure 1:**
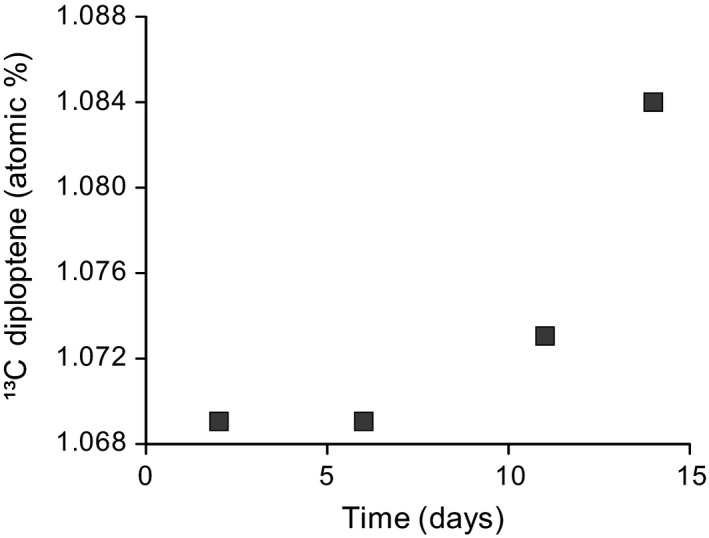
In situ pulse‐chase experiment showing ^13^C incorporation (in atomic %) into the bacterial lipid diploptene through time, after injection of ^13^C‐labelled methane into the peat. Compound‐specific carbon isotopes were measured on the lower parts of the pool‐derived *Sphagnum*, where potential methane oxidation rates were highest (van Winden, Reichart, et al., 2012)

To establish in situ methane uptake by methanotrophs and verify methanotroph‐derived lipids, an isotopic pulse‐chase experiment using ^13^C‐labelled methane was performed in the field. Within 14 days, a shift of 0.015% in ^13^C, or 15‰ in δ^13^C, was observed in diploptene, the most abundant hopene in the lower parts of the pool‐derived *Sphagnum* (Figure 1). The magnitude and evolution of the shift in ^13^C indicate uptake of the ^13^C‐labelled methane and, hence, evidence for diploptene as marker for methanotrophs.

**Figure 2:**
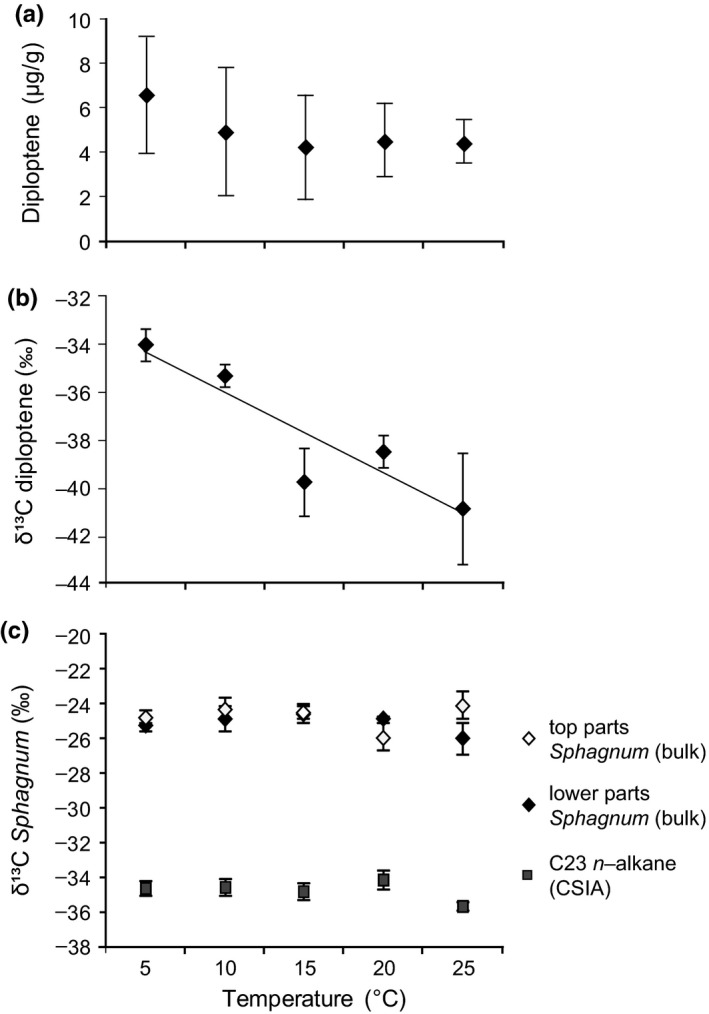
Response of potential methanotrophy proxies on temperature (°C) in the two‐month peat mesocosm incubation study. (a) Diploptene concentrations (µg/g dry weight) in the lower parts of *Sphagnum* mosses, with error bars representing ± standard errors. (b) The δ^13^C values of diploptene (‰ vs. VPDB) in the lower parts of *Sphagnum* mosses. (c) Bulk δ^13^C values (‰ vs. VPDB) of newly grown top parts and of lower parts of *Sphagnum* sp. and compound‐specific δ^13^C values of the C_23_ *n*‐alkane measured for newly grown top parts of *Sphagnum* sp. All δ^13^C values represent the means of three replicate incubations and the standard deviation is indicated

**Figure 3:**
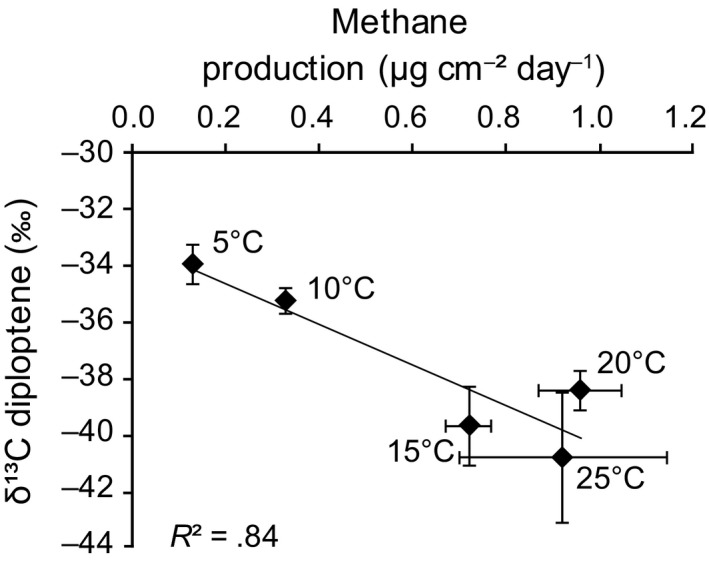
Compound‐specific δ^13^C values of diploptene (in ‰ vs. VPDB) versus the rate of methane production (μg cm^−2^ day^−1^), that is, the diffusive methane flux without the oxidative *Sphagnum* layer which was removed shortly prior to the flux measurements. Data on methane production are from van Winden, Reichart, et al. (2012). Compound‐specific δ^13^C values are means of three replicate incubations. The standard deviations indicated are based on replicate incubations

To study potential methanotrophy proxies, intact peat cores containing living *Sphagnum* spp. obtained from the field were incubated at 5, 10, 15, 20 and 25°C for two months. We have previously demonstrated that with increasing temperature, methane production and consumption (presumably through methanotrophy) increases (van Winden, Reichart, et al., 2012). Therefore, this set of experiments is useful to evaluate biomarker proxies for methanotrophy. The concentrations of diploptene were highest in the lower parts of the *Sphagnum* mosses obtained from the mesocosm temperature experiments but did not vary significantly (*p* > .01) with increasing incubation temperature (Figure 2a). However, the δ^13^C values of diploptene showed a strong negative relation with temperature (*p* < .01, *R* ^2^ = .84, Figure 2b), from −33.9‰ (at 5°C) to −40.7‰ (at 25°C). A strong correlation between methane production and diploptene δ^13^C values (*p* > .01, *R* ^2^ = .84; Figure 3) was also observed.

**Figure 4:**
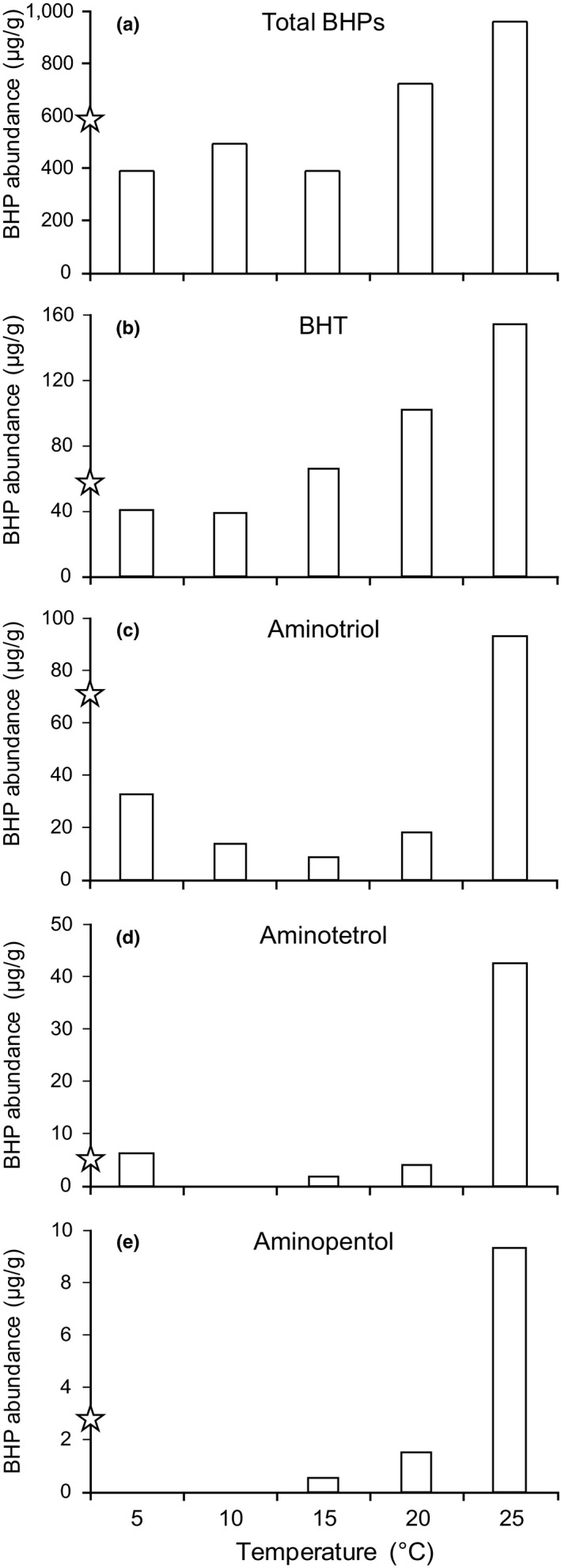
The summed BHP abundances (a) and the abundances of (b) BHT, (c) aminotriol, (d) aminotetrol and (e) aminopentol, specific BHPs produced by methanotrophs (b–e), in the lower parts of the *Sphagnum* sp. of the mesocosm experiments versus incubation temperature (°C). All concentrations are expressed in μg/g dry weight. Note the differences in scale. The accuracy of the concentrations is ±20%. Stars indicate the average BHP abundance in the middle and lower parts of *Sphagnum* sp. mosses as determined in fresh field samples (data from van Winden, Talbot, Kip, et al., 2012). The summed BHP concentrations do not reflect the summed concentration of the BHPs shown in panels b–e, as the most abundant BHPs, BHT pentose, BHT cyclitol ether and adenosylhopanes, are BHPs not produced by methanotrophs (see text) and therefore not shown here

The summed BHP concentration in *Sphagnum* showed an increase with increasing temperature upon incubation (Figure 4a). The concentrations of bacteriohopanetetrol (BHT) showed consistently increasing concentrations with increasing temperature from 10°C onwards (Figure 4b), while those of aminotriol did not increase until 25°C (Figure 4c), where a sudden fourfold increase was noted. Concentrations of aminopentol and aminotetrol, both biomarkers for methanotrophs (van Winden, Talbot, Kip, et al., 2012, and references therein), also showed this strong non‐linear response at 25°C (Figure 4e–d). Aminotetrol concentrations were almost five times higher than those of aminopentol (Figure 4). Concentrations of summed BHPs in *Sphagnum* cores incubated at 5, 10 and 15°C were lower compared to natural field‐derived *Sphagnum* samples, while those incubated at 20 and 25°C showed higher concentrations (Figure 4a). All reported individual BHP concentrations at 25°C were elevated compared to natural samples (Figure 4b–d). Aminotriol and aminopentol abundances in the natural *Sphagnum* samples were higher than those measured in *Sphagnum* cores incubated at 5–20°C, while BHT and aminotetrol abundances of natural samples were more in line with observations under experimental conditions in the range 5–20°C.

Both top and lower parts of *Sphagnum* yielded bulk δ^13^C values of around −24‰ to −26‰ that did not vary with incubation temperature (Figure 2c). Also, the C_23_ *n*‐alkane, extracted from newly grown top parts of *Sphagnum*, exhibited no significant variation in δ^13^C values with temperature; they ranged from −34‰ to −36‰ (Figure 2c).

## 4 DISCUSSION

### 4.1 BHP distributions as potential proxies for methanotrophy in ancient peats

The increase in the summed BHP concentration in *Sphagnum* moss with increasing temperature (Figure 4a) indicates that bacterial BHP production may have had a strong response to the increased temperature and associated increase of the methane flux and anticipated level of methanotrophy (cf. van Winden, Reichart, et al., 2012). Aminopentol and aminotetrol, quite specific BHPs for methanotrophs (van Winden, Talbot, Kip, et al., 2012 and references therein), showed a strong non‐linear response to the increase in temperature/methane flux, only displaying relatively high abundances at 25°C (Figure 4d,e), suggesting that their abundances do not linearly correlate with the anticipated methanotrophic activity. Osborne et al. (2017) also showed a non‐linear 10‐fold increase in the aminopentol concentration with increasing temperature (from 4 to 40°C) in methane‐amended aerobic river‐sediment incubations with only marginal increases in aminotriol and aminotetrol concentrations. These changes were most likely the effect of temperature since Sherry, Osborne, Sidgwick, Gray, and Talbot (2016) showed that changes in methane concentrations alone did not result in changes of the microbial community. This suggests that BHP production may have increased to ensure membrane integrity in the incubations at 25°C, which is a relatively high temperature for the *Sphagnum‐*inhabiting bacteria. However, Doughty, Hunter, Summons, and Newman (2009) reported that bacteria in a vegetative state produce more BHPs than when active, implying that BHP abundance does not necessarily reflect bacterial activity. This potentially complicates the use of BHPs as a palaeoenvironmental proxy for methanotrophic activity.

Contrary to our previous study on fresh material from the same site where aminotetrol and aminopentol displayed equal abundances (van Winden, Talbot, Kip, et al., 2012), here aminotetrol concentrations were almost five times higher than those of aminopentol (Figure 4d,e). Aminotetrol is primarily produced by type II methanotrophs, but also, to a minor extent, by type I methanotrophs and some sulphate‐reducing bacteria (Blumenberg et al., 2006; Neunlist & Rohmer, 1985b; Rohmer et al., 1984; Talbot et al., 2001; van Winden, Talbot, Kip, et al., 2012). Considering the low sulphate levels in peat bogs, sulphate‐reducing bacteria are not expected to be abundant in these settings; hence, type II methanotrophs and, to a lower extent type I, methanotrophs are expected to be the predominant producers of aminotetrol here. Therefore, most likely, type II methanotrophs were more prevalent than type I methanotrophs in our incubation experiments. This could be caused by experimental conditions being more favourable for the production of aminotetrol by type II methanotrophs, and/or these methanotrophs were better able to adapt to the perturbation. Still, some caution should be taken with this interpretation, as Osborne et al. (2017) and the results of our study demonstrate that the bacterial BHP production in response to environmental conditions is non‐linear. Furthermore, Osborne et al. (2017) found that aminotriol production stabilized after 10 days, while aminotetrol and aminopentol concentrations still increased with time, even after 28 days. Our incubation experiment lasted for 2 months, but it is possible that the duration of the experiment may have been insufficient for the methanotrophic community to fully adapt and express the effect of temperature on bacterial growth and BHP production.

Abundances of aminotetrol and aminopentol were small relative to the summed BHP abundance (Figure 4). This suggests that methanotrophs represent only a minor fraction of the BHP‐producing bacterial community. In fact, the most abundant BHPs in *Sphagnum* mosses are unsaturated BHT pentose, BHT pentose, BHT cyclitol ether and adenosylhopanes, which are not produced by methanotrophs (van Winden, Talbot, Kip, et al., 2012). Not all type II methanotrophs produce aminotetrol, and this trait appears to be confined to type II methanotrophs belonging to the *Methylocystaceae* family. *Methylocella*‐like type II methanotrophs only produce BHT and aminotriol (van Winden, Talbot, Kip, et al., 2012; Talbot et al., unpublished data). Aminotriol and BHT are significantly more abundant in *Sphagnum* compared to aminotetrol and aminopentol (Figure 4), and while the BHT concentration follows the same pattern as the summed BHP concentration, the aminotriol abundance shows a close resemblance to the patterns of aminotetrol and aminopentol (Figure 4c–e), suggesting that it is also produced by *Methylocella*‐like type II methanotrophs. Hence, the abundance of methanotrophs may be considerably higher than what would be estimated based on aminotetrol and aminopentol abundances alone. Still, as only aminotetrol and aminopentol are relatively specific for methanotrophs, we will have to rely on these for palaeoenvironmental reconstructions on ancient peats.

Analyses of compound‐specific isotopes of intact BHPs would enable a tremendous step forward in the understanding of their sources. Recently, a novel method was proposed to measure the ^13^C composition of BHPs, using semi‐preparative ultrahigh pressure liquid chromatography followed by high‐temperature gas chromatography–isotope ratio mass spectrometry (Hemingway et al., 2018). This method, or high‐temperature gas chromatography, as recently developed by Lengger et al. (2018), would allow this and may provide more insight into the use of methanotroph‐derived BHPs.

The methanotroph BHP markers aminotetrol and aminopentol have been detected in peat cores from Misten Bog, Belgium (van Winden, Talbot, De Vleeschouwer, Reichart, & Sinninghe Damsté, 2012) and in a peat core from Bissendorffer Moor, Germany (Talbot, McClymont, et al., 2016), where in both cases aminotetrol was more abundant than aminopentol, in agreement with our results (Figure 4). Aminotetrol and aminopentol were even present in the 55 Ma old Cobham lignite (Talbot, Bischoff, Inglis, Collinson, & Pancost, 2016), indicating that these BHPs can be used to trace back methanotrophy in ancient peats. Their occurrence reveals the past presence of methanotrophic bacteria of type I and/or II, where changes in the relative distributions point towards changes in methanotrophic communities. Hence, they potentially provide useful information to assess methanotrophic community structures in past environments, especially in the absence of other information sources such as DNA and unsaturated fatty acids.

### 4.2 Diploptene δ^13^C as a potential proxy for methanotrophy in ancient peat

The field pulse‐chase experiment using ^13^C‐labelled methane clearly showed label incorporation into diploptene by the magnitude of the isotopic shift of 0.015% ^13^C or 15‰ δ^13^C (Figure 1). Therefore, these results confirm the in situ methanotrophic activity in *Sphagnum* under natural conditions (Kip et al., 2010; Raghoebarsing et al., 2005) and make it likely this also occurs in the mesocosm experiments performed in this study. The amended ^13^C‐labelled methane represented only a fraction of the total available methane and is also not homogenously transported through the contained *Sphagnum* and peat. It is, therefore, not surprising that the isotopic shift in the δ^13^C of diploptene is not larger than the 15‰ observed. This label uptake in diploptene, which is in line with previous laboratory studies (van Winden et al., 2010), confirms its, at least partial, methanotrophic origin (Rohmer et al., 1984). Moreover, these results show a direct and fast response of the δ^13^C of diploptene to changes in the δ^13^C of the methane. In the studied *Sphagnum* moss, diploptene (hop‐22(29)‐ene) was the most abundant C_30_ hopene, while in other *Sphagnum* mosses or peats, its precursor lipid, diplopterol (Raghoebarsing et al., 2005) or hop‐17(21)‐ene (van Winden et al., 2010), was to be the most abundant C_30_ hopanoids. Probably, hop‐17(21)‐ene is the isomerization product of diploptene, which might be readily formed in the acidic peat environment (van Winden, Talbot, De Vleeschouwer, et al., 2012).

The concentrations of diploptene in the *Sphagnum* moss from the mesocosm experiments did not show significant changes with increasing temperature (Figure 2a), suggesting no major increase in biomass of the associated methanotrophs. In contrast, diploptene δ^13^C values showed a strong correlation with temperature (Figure 2b). Consequently, there is also a strong correlation of diploptene δ^13^C values with methane production (Figure 3), which has previously been established by measurement of the methane flux from the *Sphagnum* cores when the methanotroph‐containing *Sphagnum* layer was removed (van Winden, Reichart, et al., 2012). If diploptene δ^13^C values would be controlled by temperature directly, one would expect an opposite trend than observed (Figure 2b); at higher temperatures, chemical reactions are faster and isotopic discrimination is expected to decrease with increasing temperature (Hayes, 2001). This was, for example, observed by Jahnke, Summons, Hope, and Des Marais (1999) in their study of the effect of growth temperature on isotopic fractionation in methanotroph cell cultures. Therefore, the decreasing δ^13^C values of diploptene could be interpreted as being caused by the enhanced methane flux related to the increase in temperature. The decreasing δ^13^C values of diploptene could, thus, be caused by an increased contribution of ^13^C‐depleted methanotroph‐derived diploptene to the total bacterial diploptene pool. The trend of more depleted δ^13^C values with increasing methanotrophic activity is, however, not supported by diploptene concentrations, which did not change significantly with temperature (Figure 2a). Indeed, the concentrations of methanotroph‐specific BHPs also did not show a clear temperature response (Figure 4c–e), except at 25°C. Furthermore, the methanotrophs represented only a small fraction of the BHP‐producing bacterial community as revealed by our BHP results (Figure 4). Hence, it is unlikely that the shift of almost 7‰ in diploptene δ^13^C values is caused by an increased activity of methanotrophs. However, the percentage of methanotroph‐derived BHPs does not necessarily directly translate for the proportion of methanotroph‐derived diploptene. BHPs and diploptene may have different roles within the cell, and/or diploptene could be an intermediate product, which could be the reason for a lack of build‐up (Bradley, Pearson, Sáenz, & Marx, 2010). In addition, as discussed, aminopentol and aminotetrol abundances may significantly underestimate the size of the methanotrophic community.

Despite these considerations, an alternative explanation should be considered. It may well be that substrate availability influenced the extent of fractionation and, thereby, diploptene δ^13^C values. Jahnke et al. (1999) showed that δ^13^C values of methanotroph lipids strongly depend on methane concentrations, with much stronger isotopic fractionation at higher methane concentrations. An increase from 3% to 37% methane in the headspace of their culture experiments resulted in up to 22‰ more negative δ^13^C values of total methanotroph lipids. Therefore, the decrease in diploptene δ^13^C values with increasing methane availability (Figure 3) can also be explained through enhanced expression of the enzymatic isotope effect associated with methanotrophy, which was suppressed by substrate or mass transport limitations at lower temperatures or lower methane concentrations (Hayes, 2001).

Unfortunately, we did not analyse the isotopic composition of methane and carbon dioxide during the mesocosm experiments, which could have further assisted in the interpretation of the results. In addition, it would be recommended to perform a set of mesocosm experiments with *Sphagnum* moss where the methane concentration is varied, while the temperature is kept constant, to decouple the temperature effect from the methane concentration effect, similar to the experiment performed by Sherry et al. (2016) and Osborne et al. (2017).

Diploptene and hopane δ^13^C values have been used previously to trace methanotrophy in past peatlands. In a *Carex*‐dominated Holocene peat deposit from the Tibetan Plateau, diploptene δ^13^C values as negative as −50‰ have been reported (Zheng et al., 2014). In a recent study by Huang et al. (2018) in a peatland in Central China, the δ^13^C values of 17β,21β(H)‐norhopane ranged between −28‰ and −40‰ and the more negative values were interpreted to be caused by enhanced methanotrophy. In both studies, the more negative hopanoid δ^13^C values corresponded to dryer conditions, which were explained to be the result of aeration of deeper peat deposits below the rooted zone, enhancing methanotrophy benefitting from an enhanced diffusive methane flux at the oxic–anoxic interface (Huang et al., 2018; Zheng et al., 2014). Extending the geological record, Inglis et al. (2015) observed no change in hopane δ^13^C values in a Paleogene peatland (Schӧningen, Germany), although there was a rise in the water table. In contrast, in the Cobham lignite, a Paleogene wetland deposit, negative δ^13^C values, extending to −42‰ and −76‰ for the C_31_ and C_29_ hopanes, respectively, were observed (Pancost et al., 2007). In the context of our data from experiments with extant peats, these depleted hopane δ^13^C values in the geological record suggest an intensified methane cycle during these key periods of Earth's past.

The environmental variability in peat bogs is much higher than represented in our limited set of experiments where we singled out diffusion‐dominated waterlogged conditions with *S. cuspidatum,* all optimal for methanotrophy. Transport pathways largely govern in situ methane concentrations, which complicate the interpretation of the diploptene δ^13^C values as palaeo‐proxies. In turn, temperature also plays a key role on the transport efficiency of methane. Still, diploptene (or hopane) δ^13^C values of ancient peats would represent a median where the variability is averaged out. The observed relationship of decreasing diploptene δ^13^C values with enhanced methane flux rates (Figure 3) is indirect and not unequivocal, still it indicates that a record of δ^13^C values of diploptene, or its diagenetic products, could be a useful proxy to assess at least relative shifts in past levels of methanotrophy.

### 4.3 *Sphagnum* bulk and compound‐specific δ^13^C values as potential proxies for methanotrophy in ancient peat

No significant variation was observed in the δ^13^C values of bulk *Sphagnum* moss and the C_23_ *n*‐alkane, the most abundant *n*‐alkane in *S. cuspidatum* and other *Sphagnum* sp. (Baas, Pancost, van Geel, & Sinninghe Damsté, 2000; Nott et al., 2000), with incubation temperature (Figure 2c) and, hence, with changes in methane production. This lack of variation in *Sphagnum* δ^13^C values suggests that *Sphagnum* does not take up more methane‐derived CO_2_ relative to CO_2_ from other sources at increased levels of methane production and anticipated methanotrophy. This is somewhat surprising since it is thought that *Sphagnum* also uses the CO_2_ produced by methanotrophs for photosynthesis (Kip et al., 2010; Raghoebarsing et al., 2005). Increased levels of methanotrophy would thus expected to be reflected in a more negative ^13^C signature of both specific lipids produced by *Sphagnum* or even in its bulk value.

Increasing temperatures could have stimulated organic matter degradation; hence, the increased methane flux at higher temperatures may be offset by a corresponding increase in the CO_2_ flux from enhanced organic matter degradation. The isotopic composition of the combined recycled carbon source for *Sphagnum* would thereby remain the same. In addition, the isotopic fractionation between CO_2_ and methane during methanogenesis may be balanced, with methane becoming more enriched in ^13^C when more is produced, while CO_2_ becomes less enriched in ^13^C when less CO_2_ is turned into methane. Such an inverse relationship between the carbon isotopic values of CO_2_ and methane has been observed in peat bogs by Hornibrook et al. (2000). This way, changes in the isotopic composition of methane and CO_2_ would cancel each other out. In any case, the lack of a clear temperature response of the stable carbon isotope values in both bulk *Sphagnum* and *Sphagnum‐*derived biomarkers implies that reconstruction of methane oxidation and methane (re)cycling in peat bogs will have to rely on biomarkers specific for methanotrophs, such as δ^13^C values of hopenes and specific BHPs.

## 5 CONCLUSIONS

A field pulse‐chase experiment using ^13^C‐labelled methane clearly demonstrated ^13^C incorporation into diploptene and hence support in situ methanotrophic activity in *Sphagnum* under natural conditions. Mesocosm experiments with *Sphagnum* peat cores incubated at different temperatures resulted in large variations in the δ^13^C values of diploptene extracted from *Sphagnum,* with values of −34‰ at 5°C and −41‰ at 25°C*.*

The diploptene δ^13^C values showed a strong correlation with temperature and methane production, while concentrations of diploptene did not vary significantly. The diploptene δ^13^C values can be explained by elevated methane availability at higher temperatures, resulting in enhanced expression of the enzymatic isotope effect. The δ^13^C values of bulk *Sphagnum* or *Sphagnum*‐derived C_23_ *n*‐alkane did not show any significant variation with temperature. The lack of any trend implies that reconstruction of methane oxidation and methane (re)cycling in peat bogs will have to rely on biomarkers specific for methanotrophs, such as δ^13^C values of hopenes and specific BHPs. Methanotroph‐specific BHPs, aminotetrol and aminopentol, showed a non‐linear response to temperature. Aminotetrol was more abundant compared to aminopentol, which indicates that type II methanotrophs became prevalent during the experiment. Relative BHP concentrations may therefore provide insight into the presence of different types of methanotrophs in ancient peats. Finally, the δ^13^C values of diploptene, or its diagenetic products, potentially provide a useful tool to assess methanotrophic activity in past environments.

## CONFLICT OF INTERESTS

The authors declare that they have no conflicts of interests.
